# A Threat Explained

**Published:** 1845-03

**Authors:** 


*A Threat Explained.*—"Your unchristian virulence against me," said a Huguenot, who had been persecuted for preaching, "shall cost hundreds of people their lives." This menace brought the author into trouble; he was cited to a court of justice, and was charged with harboring the most bloody designs against his fellow-subjects. "I am innocent," said he, "of all you lay to my account, my only meaning was, that I intended (since I could not act as a minister) to practice as a physician." If the Huguenot had practised, without the requisite knowledge, he would have kept his promise.

